# Bed for three

**DOI:** 10.1192/j.eurpsy.2021.2032

**Published:** 2021-08-13

**Authors:** C. Vallecillo Adame, T. Jiménez Aparicio, C. De Andrés Lobo, M. Queipo De Llano De La Viuda, G. Guerra Valera, A. Gonzaga Ramírez, I. Santos Carrasco, J. Gonçalves Cerejeira, C. Capella Meseguer, E. Rodríguez Vázquez

**Affiliations:** 1 Psiquiatría, Hospital Clínico Universitario Valldolid, Valladolid, Spain; 2 Psiquiatría, Hospital Clínico Universitario Valladolid, Valladolid, Spain; 3 Psiquiatría, HCUV, Valladolid, Spain; 4 Psiquiatría, Hospital Clínico Universitario de Valladolid, Valladolid, Spain

**Keywords:** Charles Bonnet, Hallucinations, retinal detachment

## Abstract

**Introduction:**

Charles Bonnet syndrome (CBS) is characterized by the presence of visual hallucinations without other sensory-perceptual disturbances or evidence of organic mental disorder nor functional psychosis.

**Objectives:**

Review differential diagnosis of BCS, searching articles in Pubmed.

**Methods:**

62-year-old woman, undergoing treatment with Sertraline and psychotherapy for three months because of anxious-depressive synthoms. Pathological myopia and retinal detachment in 2012, blind left eye, retaining 33% vision in the right eye. She comes to the emergency room feeling really anxious, she says that for a year now she has had the feeling that her husband is cheating on her with another woman, and she claims with certainty that she sees a woman in her bed at night, as well as flashes of light evidencing her presence. She has also begun to hear voices through the telephone wires. She and her family deny memory loss or other cognitive impairments. We performed a Nuclear Magnetic Resonance with normal results. Family claims good conygal relation until these synthoms began and no signs of cognitive impairment.

**Results:**

The patient lives as real these hallucinations which haven´t appeared during admission. We started treatment with an antipsychotic and a benzodiazepine, with great improvement of anxiety and development of some insight. Executive impairment was observed.

**Conclusions:**

The results obtained, make us think that, although our patient has an important visual loss, it is more a psychiatric pathology. Here lies the importance of a multidisciplinary approach among ophthalmologists, neurologists and psychiatrists in order to avoid misdiagnosis and that the patient can benefit from proper treatment.

**Disclosure:**

No significant relationships.

